# Are STEM Students Creative Thinkers?

**DOI:** 10.3390/jintelligence11060106

**Published:** 2023-06-01

**Authors:** Christabel Borg Preca, Leonie Baldacchino, Marie Briguglio, Margaret Mangion

**Affiliations:** 1 Department of Public Policy, University of Malta, MSD2080 Msida, Malta; 2 The Edward de Bono Institute for Creative Thinking and Innovation, University of Malta, MSD2080 Msida, Malta; 3 Department of Economics, University of Malta, MSD2080 Msida, Malta

**Keywords:** STEM, creativity, divergent thinking, 21st century skills, secondary education

## Abstract

Scholarly research has increasingly examined the role of STEM (Science, Technology, Engineering, and Mathematics) education, and that of creativity as a transversal skill. However, far fewer studies have investigated the relationship between the two, particularly in secondary-school contexts, and they have obtained inconsistent results. This paper contributes to the literature by asking: *To what extent is studying STEM associated with higher levels of creativity in a secondary-school context?* The study utilises a pre-existing dataset gathered in Malta (EU) from some 400 students aged between 11 and 16 years old. It yields information on both the engagement in STEM (measured by exposure to STEM chosen by students as optional subjects, and the enjoyment of STEM considered by students to be their favourite subjects), as well as creativity levels (measured by Divergent Thinking performance on Alternate Uses Tests). Correlation analysis revealed a strong positive link between the two phenomena, lending support to the notion that STEM students tend to be more creative than other students. Using regression analysis, a model is estimated to identify the possible effects of engaging in STEM subjects on creativity, once the other co-determinants of creativity are controlled. The results indicate that both the exposure to STEM subject/s and enjoyment thereof significantly and positively predict creativity, even after controlling for the other possible determinants of creativity (such as age, gender, parental education, and participation in creative activities). These findings offer encouraging insights into 21st century education and for curriculum development as they suggest that, in addition to having value in their own right, STEM subjects can contribute to the development of creativity in young people.

## 1. Introduction

In recent years, there have been considerable efforts among educators and policymakers to promote science, technology, engineering, and mathematics (STEM) subjects and careers, as they are considered by many to play a key role in boosting economic competitiveness, productivity, and innovation (Collard and Looney 2014; European Commission 2009; Runco 2010). STEM education is often linked to economic competitiveness, as it is believed to contribute to workforce-ready individuals (Bentley et al. 2022). Indeed, STEM-related jobs are among the fastest-growing jobs in the labour market. Within the European Union (EU), the number of people working in STEM-related fields increased by 12% between 2003 and 2013. To date, STEM accounts for 7% of the total occupations within the EU. STEM occupations are also among the highest-paying sectors (Melguizo and Wolniak 2012) and are associated with improved economic, social, and personal wellbeing (Beede et al. 2011). 

In parallel, there has been an increasing understanding of the importance of creativity as a transversal 21st century skill (Partnership for 21st Century Learning 2015; Voogt and Roblin 2012). Creativity typically refers to the generation of ideas that are novel, original, or unique, as well useful, appropriate, or contextually relevant (Amabile et al. 1996; El Murad and West 2004; Runco and Jaeger 2012). Creativity is increasingly considered to be a crucial competency (Marrone et al. 2022) for economic and social development. Yet, while STEM and creativity are both observed as important skill sets, a question remains as to whether one comes at the cost of the other. 

Traditionally, the Arts and Humanities were considered to be linked to creativity and, in particular, divergent thinking (DT), which involves the generation of multiple ideas or solutions related to a task or situation (Cropley 2006). The Sciences, on the other hand, tend to be associated more with convergent thinking, in the sense of tackling problems by providing a single optimal solution (Cropley 2006). Indeed, during his influential ‘Rede Lecture’, titled ‘The Two Cultures’, in 1959, scientist and novelist C.P. Snow highlighted a division between the Humanities and Sciences. This has since triggered scholarly curiosity as to whether there is a difference in thinking styles across domains (Furnham et al. 2011). 

A number of researchers have since sought to examine the links between STEM subjects and creativity, with inconsistent results. Studies by Furnham et al. (2011), van Broekhoven et al. (2020), and Williamson (2011) all indicate that there are no differences in creative thinking abilities between students from different disciplines. Stylianidou et al. (2018), on the other hand, suggest that science education in the early years may enhance creativity. There therefore remains an unresolved question regarding whether there is any relationship, positive or negative, between engaging in STEM education and creativity. 

## 2. Theoretical Background 

### 2.1. STEM Education

STEM education is a complex and poorly defined field (Li et al. 2020) that has been the focus of an increasing body of research since 2000 (Kayan-Fadlelmula et al. 2022; Li et al. 2020; Li and Xiao 2022). What started as an acronym, bringing together the four core subjects of Science, Technology, Engineering, and Mathematics, has changed considerably over the years (Dare et al. 2019; Martín-Páez et al. 2019), to the extent that Breiner et al. (2012) argue that STEM education holds different meanings to different stakeholders. Tytler (2020) notes that the STEM acronym is underpinned by the notion that it collectively comprises “a coherent package of subjects that cover the knowledge and skills around the sciences, applied sciences, and the digital world that constitute the driving force towards a post-industrial global future and the future wealth of countries” (p. 22). 

Of particular interest to this study is the teaching of STEM in secondary-school contexts, where different approaches to STEM education have been observed. Kennedy and Odell (2014) identify a number of STEM initiatives within the US education system, targeting students from secondary-level education to high-school-level (14–18 years old) students. These include the T-STEM programme that targets students from 6th to 12th grades, providing a hands-on approach to STEM education and linking students with industry. This programme has not only been associated with greater academic success, but also with fewer reports of disruptive behaviour. Reiss and Mujtaba (2017) highlight that students who are exposed to careers education in STEM, such as increasing their awareness of the transferability of STEM skills or the higher income associated with STEM careers, have an increased likelihood that they will choose STEM subjects at a post-secondary-education level. However, Kennedy et al. (2020) show that students’ attitudes towards STEM subjects, and their likelihood of pursuing STEM subjects at a post-secondary level, vary greatly between the different subjects, depending on their perceived relevance, personal usefulness, enjoyability, and self-efficacy.

By any definition, STEM education is widely believed to hold great potential for teaching students how to tackle complex problems that dominate our world, such as climate change, the depletion of natural resources, and public health crises (Merrill and Daugherty 2010). It enables students to develop skills in communication, critical thinking, problem-solving, and the analysis of data, while developing an understanding of processes and provision of solutions based on knowledge (Froschauer 2015; Jang 2016). Kennedy and Odell (2014) highlight how engineering can add a dynamic element to the teaching of Science and Mathematics that promotes STEM literacy, defined as the ability to engage with and communicate complex problems with audiences. Consequently, STEM is considered a critical domain of knowledge that is linked to the success of students and graduates in the 21st century (Sanders 2009).

### 2.2. Creativity Education

At the same time, there is broad agreement in academia and industry about the need for skills that are transversal, multidimensional, and associated with higher-order cognitive processes, in order to deal with problems that are complex and diverse (Brown et al. 2010). Foremost, among such skills is creativity (Partnership for 21st Century Learning 2015). A number of studies focus on nurturing creativity in educational contexts. For instance, Briguglio et al. (2022) identify the impact of a school-based educational programme in secondary-school contexts, finding that, while creativity levels generally decreased between the start and end of the school year, students exposed to a programme with creative practitioners fared better. The same study also found that parental education has a positive effect on creativity. 

A key theme in the literature is the criticism of educational systems for inadequately nurturing transversal skills, particularly creativity (Beghetto and Madison 2022; Darbellay 2022). Corazza et al. (2021) refer to the “Traditional Education System (TES)” where creativity is not only omitted as an explicit objective, but students even tend to be penalised for behaviour associated with creativity, such as mind-wandering, slow responses, or unexpected answers. The authors argue that “creativity … has today become a democratic necessity” and that “there is today an urgent call for the formal introduction of creativity inside the education system” (p. 72). Yet, it seems safe to say that the introduction of creativity to the education system is generally perceived as an unwelcome disruption and met with resistance. In view of this, Beghetto and Madison (2022) maintain that “it is not students who need to get smarter before they address complex challenges, rather, it is schools that need to get smarter in the kinds of curricular opportunities they provide young people” (p. 11).

### 2.3. The Relationship between STEM and Creativity 

While there has been considerable scholarly attention paid to both STEM and creativity separately, there is a wide scope to examine the link between the two. Learning methods and thinking styles that prevail in the Sciences have traditionally been believed to differ from the ones in the Arts and Humanities. Specifically, individuals who are attracted to the Sciences are considered to prefer convergent thinking, while those who gravitate towards the Arts and Humanities are assumed to prefer creativity and DT (Furnham et al. 2011). However, empirical studies have contradicted these assumptions. For example, Williamson (2011) found only minor differences in learning styles and no significant differences in problem-solving behaviour among undergraduate students from different domains. Furnham et al. (2011) also found no significant differences in DT fluency, creative judgement, or achievement between Arts and Science undergraduate students. Similar results were obtained by van Broekhoven et al. (2020) who found no significant differences in creativity between university students specialising in the Arts or STEM-related fields. 

Furthermore, some authors (e.g., Root-Bernstein et al. 2008) suggest that there is actually a positive link between scientific success and ability within artistic domains, as noted in polymaths who excel in a variety of disciplines, including the Sciences and Arts. Indeed, it may also be argued that creativity can be developed through STEM subjects (Zhbanova 2019). Sánchez et al. (2022) consider creativity to emerge from processes that are essential in Science and Mathematics activities. This suggests that STEM may play an indirect role in the development of creativity by facilitating these processes. In support of this, Schoevers et al. (2020) found that mathematical ability and the ability to generate ideas influenced mathematical creativity among fourth graders. Stylianidou et al. (2018) also found that the focus on problem-solving activity and agency in STEM has a positive impact on student creativity. 

Conversely, some authors focus on the effect of creativity on STEM. Henriksen (2014) argues that the link between creativity and the Arts is evident in cases of successful leaders and innovators. Lubart et al. (2022) argue that creativity is fundamentally important in Science, and note that many scientific breakthroughs are the product of creative thinking. Peppler and Wohlwend (2018) note that art and creativity can facilitate STEM engagement by making these subjects appear more approachable and relatable.

It is worth noting that much of the research on the relationship between STEM and creativity in educational settings has been at a tertiary level (van Broekhoven et al. 2020; Furnham et al. 2011; Williamson 2011), possibly because of a greater distinction of the subjects studied and greater clarity concerning the choice of specialisation at this level. 

### 2.4. Synthesis, Research Question, and Hypotheses

In summary, there seems to be little disagreement in the literature that both STEM subjects and creativity are important skills to be nurtured in an educational context. However, the results are inconsistent as to whether there is a positive effect of nurturing creativity for STEM performance and whether there is a positive effect from engaging in STEM on creativity itself. Moreover, the extant research on the relationship between STEM and creativity in educational settings has largely occurred at post-secondary-, tertiary-, and, to a lesser extent, early school settings, with far less evidence being obtained from a secondary-school level (pre-college age). 

Against this backdrop, this study focuses on the first relationship and asks the following research question: To what extent is studying STEM associated with higher levels of creativity in a secondary-school context? More specifically, we set out to assess, against the null hypothesis (H0) of zero association, the alternative hypothesis that students who learn STEM subjects demonstrate a higher level of creativity, as measured by DT. We test this hypothesis in two ways: firstly by assessing whether students who choose STEM subjects demonstrate greater creativity, thereby capturing the effect of exposure to STEM subjects (H1a), and secondly, by assessing whether students who consider STEM subjects to be their favourite subjects exhibit higher levels of creativity, thereby capturing the effect of the enjoyment of STEM subjects (H1b). We test our hypotheses in the milieu of a Maltese secondary-school environment. This context and the method adopted are described in the following section. 

## 3. Materials and Methods

### 3.1. Context of the Study

The context of this study was Malta, a European Union (EU) small island state with a population of approximately 500,000 people. Compulsory schooling in Malta is between the ages of 5 and 16 years old, and students may attend one of three types of schools, namely, state, church, and independent schools. State schools attract a particular geographic catchment, as stipulated by the boundaries set for the different districts, while church and independent schools attract students from all over the island. Although there are variations in the management and teaching policies across the types of schools, they are all required to follow the same curriculum with a generally homogeneous teaching practice, as regulated by the National Education Act (CAP 327). 

At the end of Year 8, students (aged 11–13 years) choose a number of optional academic, vocational, or applied subjects that would lead them to a Matriculation and Secondary Education Certificate (MATSEC)—an entry requirement for post-secondary education. At this time, they continue to study core subjects (English, Maltese, Mathematics, Religious Studies/Ethics, and Personal, Social, and Career Development), and they supplement these with optional subjects. These optional subjects include a range of academic, vocational, and applied subjects. Among them, and of particular interest to this study, were those identified as STEM subjects by the Maltese authorities (European Schoolnet 2018), namely, Health and Social Care, Engineering, Technology, Information Technology, Computing, Design and Technology, Biology, Chemistry, Physics, Mathematics, Graphical Communication, and Agribusiness. All schools in Malta offer STEM-related subjects at the secondary level. 

### 3.2. Data Collection

To address the research question, this study performed a statistical analysis of pre-existing survey data that were gathered in 2016 as part of a larger research project commissioned by the Arts Council Malta (see also Briguglio et al. 2022). Although a few years have elapsed since the data were gathered, there have been no major changes to the curriculum related to STEM or creativity since then. The topics remain highly relevant to stakeholders in Malta, including educators, researchers, and policymakers. A subset of the data collected was suitable to test this study’s hypotheses and was shared with the present authors, following approval by the Arts Council Malta. An overview of the original data collection procedure is provided below for the sake of completeness. 

The research sample was that of 400 students, broadly and evenly distributed by gender and aged between 11 and 16 years. The students were recruited from nine secondary schools in Malta (five state, two church, and two independent schools), all of which were originally identified by the Arts Council on the basis that they were beneficiaries of a state-funded creativity programme, they represented different school types, and they agreed to participate in the research. Participation by students in the study was voluntary and entailed the signing of consent forms by their legal guardians. Teachers were provided with written and verbal instructions to ensure that the data collection instruments were administered in a consistent manner. The original data collection was covered by ethics and data protection clearances, and permission to use the unpublished secondary data as well as ethics and data protection clearances were obtained by the present authors. 

The data were gathered by means of a paper-based questionnaire, distributed by teachers to students in their classrooms at the start of the school year, in October 2016. It is pertinent to note that this constituted an initial round of data collection prior to the implementation of the above-mentioned creativity funding programme. It took students an average of 20 min to complete the entire questionnaire. The content of the questionnaire included, inter alia, i. measures of creativity, ii. measures of engagement in STEM, iii. measures of engagement in creative activities, and iv. demographic and other data. 

As in other studies, creativity was operationalised by measuring the capacity to produce new ideas through DT—a key indicator of creativity (Doron 2016). In this regard, Guilford et al.’s (1960) Alternate Uses Test (AUT) was employed, involving the generation of multiple ideas or solutions to a given task or problem. Two such tests were assigned at the very beginning of the questionnaire, where participants were asked to think of as many uses as they could for a sock and a box, within a time limit of three minutes per object. 

The data related to STEM studies were gathered in the original survey by asking students the following questions: ‘Which optional subjects have you chosen at school? (circle any)’ (to measure exposure to STEM subjects) and ‘Think of ALL your school subjects. Which is your favourite?’ (to measure enjoyment of STEM subjects). The questionnaire also gathered data related to the participants’ background, such as age, gender, and parental education, as these were identified in the previous research as relevant co-determinants of creative outcomes (Briguglio et al. 2022; Shah and Gustafsson 2020). As a further control to understand the diversity in creativity, the questionnaire also asked students about their participation in school-based and extra-curricular creative activities. This was measured by means of an adaptation of Batey’s (2007) Biographical Inventory of Creative Behaviours (BICB), namely, limiting the options to 15 items known to be relevant in Malta (e.g., writing a short story, producing a picture, or creating a sculpture). Students were asked to mark any activities that they had been involved in. Composite scores of creative activities were then created by adding up the number of items that participants reported engaging in during school hours and during their free time in the preceding month. The number of activities chosen were subsequently collapsed into two indices—one for creative activities conducted at school and one for creative activities conducted in free time. 

### 3.3. Data Preparation and Analysis

Responses to the AUT DT test were scored by two tenured academics at a higher-education institution, knowledgeable in creativity theory, research, and practice. Indices of creativity were created, including ideational fluency (the number of ideas generated), flexibility (the number of conceptual categories into which the ideas may fit), elaboration (the amount of detail provided to communicate the ideas), originality (the uniqueness of the ideas) (e.g., Gu et al. 2019; Runco 2010; Yi et al. 2015), relevance, appropriateness or feasibility (Baruah et al. 2021), and novelty (Amabile et al. 1996; El Murad and West 2004; Runco and Jaeger 2012). Inter-rater reliability, which was estimated by Pearson’s Bivariate Correlations, was strong (*r* > .7; *p* < .001) or moderate (*r* = .5–.7, *p* < .01), and all points of divergence between the raters were resolved. 

On the basis of the data available, the researchers were able to create an index of exposure to STEM subjects and another of enjoyment of STEM subjects. There being no a priori reason to weight the STEM subjects, all of them were provided with equal weighting. To compress and simplify the data by extracting the most important information from a data table (Abdi and Williams 2010), Principal Component Analysis (PCA) was performed, thereby deriving an overall score for creativity (i.e., dependent variable) that encompassed all the above-mentioned creativity indices for both items in the DT test (sock and box). 

Table 1 presents the dataset that was used for this study. As can be observed, the 400 students were aged between 11 and 16 years with a mean age of just over 13.5 years. Just over half the sample students were female, and just over half had parents whose highest level of education was tertiary (university). Students undertook an average of 4 to 5 out of 14 creative activities at school and around 5 out of 14 possible activities in their free time. Their creativity scores ranged from 0 to 1, with a mean of 0.5. Just over two-thirds of the students chose at least one STEM subject as an optional subject with responses ranging from zero to five; however, only around one-third of the students named a STEM subject as their favourite. 

The Results Section starts with a simple description of the data, assessing the students’ engagement in STEM subjects, followed by a basic analysis by gender. A correlation analysis then followed to test the links between engagement in STEM subjects and creative activities or creativity scores. Recognising that correlation does not indicate causality (other variables may have influenced the relationship), Ordinary Least Squares (OLS) regressions were performed in order to estimate the strength of the associations between variables (Hutcheson 1999), parsing out the effect of co-determinants. There are three components of an OLS, namely, “a random component for the response variable, which is assumed to be normally distributed, a systematic component representing the fixed values of the explanatory variables in terms of a linear function, and finally, a link function which maps the systematic component onto the random component” (Hutcheson 1999, p. 2). 

In this study, the response (dependent) variable is the PCA creativity score, the explanatory (independent) variables are the STEM subjects, and the link function includes the important correlates of creativity measures (including age, gender, education level of parents, and creative activity during school hours or free time). Prior to running the regressions, the normality of the dependent variables was tested using the Kolmogorov–Smirnov test. This revealed that the measures of creativity were non-parametric. Logarithmic transformations were therefore conducted prior to using them in the regression models. The statistical analysis was performed using International Business Machines Statistical Package for Social Sciences (IBM SPSS) software.

## 4. Results

### 4.1. Preliminary Analysis

The data analysis revealed that most students in the sample had some exposure to STEM, as they chose at least one STEM subject as an optional subject (Table 2). The three most popular STEM subjects are Biology (*n* = 111), Physics (*n* = 87), and Graphical Communication (*n* = 80), whereas the least popular are Engineering (*n* = 3), Technology (*n* = 5), and Agribusiness (*n* = 6). 

While most students had some exposure to STEM, the same cannot be said regarding the enjoyment of STEM, as 64.8% of the sample (*n* = 259) did not name any STEM subjects as being their favourite subject. One third of the sample (33.7%, *n* = 135) named one STEM subject as their favourite, while only 2.5% of the sample (*n* = 6) named two or three STEM subjects as their favourite (Table 3). The most common favourite STEM subjects were Biology (*n* = 40), Mathematics (*n* = 39), and Computing (*n* = 21), while the least popular were Technology and Engineering (*n* = 0) Health and Social Care (*n* = 1), and Design and Technology (*n* = 1). 

**Figure 1:**
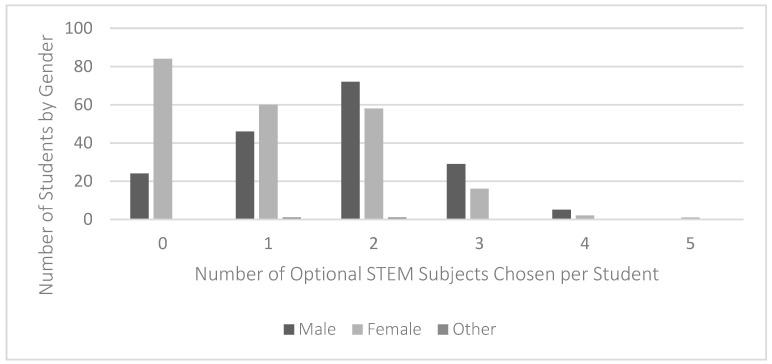
Students’ exposure to STEM subjects, distributed by gender. Note: *N* = 400.

When comparing students’ gender and exposure to STEM, it was evident that STEM subjects were more popular among boys than girls in our sample (Figure 1). Notably, many more girls (38%, *n* = 84) than boys (13.6%, *n* = 24) chose no STEM subjects as optional subjects. Similarly, more girls (27.1%, *n* = 60) than boys (26.1%, *n* = 46) chose only one optional STEM subject. Conversely, more boys (40.9%, *n* = 72; 16.5%, *n* = 29; 2.8%, *n* = 5) than girls (26.2%, *n* = 58; 7.2%, *n* = 16; 0.9%, *n* = 2) chose two, three, or four optional STEM subjects. A chi-squared test (10, *N* = 399) = 39.31, *p* < .001 confirmed that the association between gender and exposure to STEM subjects was statistically significant. 

**Figure 2:**
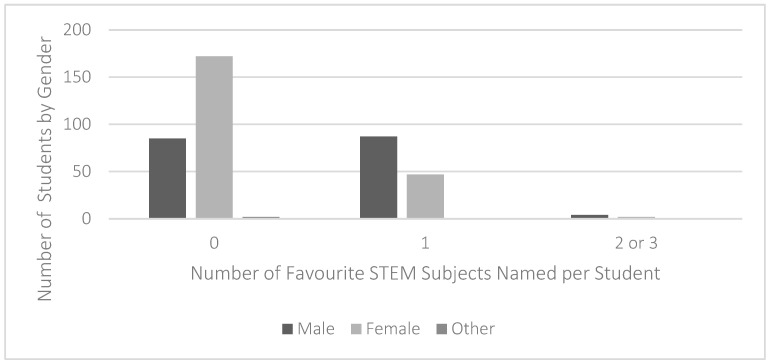
Students’ enjoyment of STEM subjects, distributed by gender. Note: *N* = 400.

When comparing students’ gender and their enjoyment of STEM subjects, it was again evident that STEM subjects were appreciated more by boys than girls (Figure 2). Most female students (77.8%, *n* = 172) named no STEM subjects as their favourite, in contrast to half the male students in the sample (49.9%, *n* = 87) who reported that their favourite subject was from the STEM category. A chi-squared test (6, *N* = 399) = 39.93, *p* < .001 confirmed that the association between gender and enjoyment of STEM subjects was statistically significant.

### 4.2. Correlation Analysis 

Since many of the variables were non-parametric, a Spearman’s Rank Order Correlation was performed. As shown in Table 4, a significant positive correlation can be noted between the engagement of students in STEM subjects (be it as a choice of optional subject or as their favourite subject) and creativity scores (measured as a PCA score). There was some support for both H1a and H1b that a positive link existed between the exposure to STEM subjects and creativity (*r* = .264, *p* < .01) and between the enjoyment of STEM subjects and creativity (*r* = .159, *p* < .01).

The correlation results, however, also indicate that there is a significant link between creativity and gender (*r* = −.185, *p* < .01), creativity and parental education (*r* = .239, *p* < .01), and creativity and participation in creative activities performed at school (*r* = .160, *p* < .01) and during free time (*r* = .211, *p* < .01). Therefore, it was difficult to determine whether the link between STEM and creativity was driven by, perhaps, a gender effect (more boys chose STEM, and boys scored better for creativity) or a parental-education effect (highly educated parents were linked to greater STEM exposure and creativity levels). For this purpose, we turned to a regression analysis.

### 4.3. Regression Analysis 

OLS regressions were performed to identify whether changes in creativity scores may be attributed to the study of STEM subjects. The variables included in the OLS models were selected on the basis of past research, including Briguglio et al. (2022). The multiple regression employed the creativity (PCA scores) as the main dependent variable in order to provide a general view of the effect of STEM subjects on overall creativity. Three models were run. The first employed a base model to forecast the creative outcomes (PCA scores). This model included three key demographic variables (age, gender, and parental education), as well as engagement in creative activities (based on the indices constructed) as predictor variables. The second model added ‘STEM Exposure’ as an independent variable while the third model replaced this with ‘STEM Enjoyment’. Table 5 shows the results of these three models. All Tolerance values were above .10, and the Variance Inflation Factor (VIF) values were less than 10, indicating that there were no multicollinearity issues.

The results displayed in column 1 show that the base model is significant (*R*^2^ = 0.120, *F* = 10.486, *p* < .001). Gender was found to be negatively associated with creativity (*B* = −0.041, *p* < .01), which implies that male students outperformed their female counterparts on the DT tests, once all other factors were controlled for. As expected from the previous literature (e.g., Briguglio et al. 2022), the level of education of the students’ parents had a positive effect on their creativity (*B* = 0.032, *p* < .001). Participation in creative activities during the students’ free time also had a significant positive effect on creativity (*B* = 0.007, *p* < .01), while participation in creative activities at school had no additional significant effect. 

The regression was repeated with the addition of ‘STEM Exposure’ as an independent variable to test H1a. This model, shown in column 2, was significant (*R*^2^ = 0.135, *F* = 9.895, *p* < .001) and was improved as a result of adding the independent variable to the base model. This independent variable was observed to have a positive effect on creativity (*B* = 0.017, *p* < .05), which indicates that the more optional STEM subjects students chose, the more likely they were to perform better in terms of creativity. This offers support for H1a. 

To further test our expectations, the regression was conducted again, this time with ‘STEM Enjoyment’ as an independent variable to test H1b. As shown in column 3, the model significance was slightly stronger than the previous model (*R*^2^ = 0.135, *F* = 9.914, *p* < .001), and it was once again an improvement on the base model. The impact of STEM as a favourite subject/s was positive and significant and twice as large as that of STEM as optional subject/s (*B* = 0.038, *p* < .05). This shows that students who indicated more STEM subjects as their favourites obtained higher creativity scores, thereby supporting H1b. 

Taken together, these findings suggest a rejection of the null hypothesis (H0) that stipulates that there is no association between the engagement in STEM learning and creativity. On the contrary, whether measured by the exposure to or enjoyment of STEM subjects, the engagement in STEM subjects was positively associated with creativity, as measured by DT. Furthermore, the results suggest that, among students who considered STEM subjects to be their favourite subjects, creativity scores are even higher. These results are derived after controlling for co-determinants of DT, including age, gender, and parental-education level, as well as engagement in creative activities at school and in their free time. 

To test for the robustness of our results, regression analyses were repeated with all the individual DT indices as dependent variables. The analysis confirmed our findings: the propensity to indicate STEM subjects as favourite subjects is positively associated with all the indices. Choosing STEM subjects as optional subjects was positively linked to elaboration (the amount of detail), originality (uniqueness), and novelty, but not significantly related to fluency (the number of ideas generated) or flexibility (the number of conceptual categories into which the ideas may fit). For ease of reference, the results are summarised in Table 6 by displaying the coefficients and respective standard errors of the main variables. 

## 5. Discussion

The main aim of this study was to explore the extent to which studying STEM subjects in secondary-school was associated with creativity levels. Although previous research has investigated the relationship between creativity and different domains of knowledge, including the Arts and STEM subjects (e.g., Furnham et al. 2011; Stylianidou et al. 2018; van Broekhoven et al. 2020; Williamson 2011), the results are inconsistent, leaving an unresolved issue regarding the relationship between STEM and creativity. Moreover, previous research on this relationship has largely focused on post-secondary- (Li and Xiao 2022), tertiary- (van Broekhoven et al. 2020; Furnham et al. 2011; Williamson 2011), and, to a lesser extent, early education levels (Stylianidou et al. 2018), leaving a gap in the knowledge concerning this question in the context of secondary-school (pre-college-age students). This study attempted to address the gap in the research by investigating the relationship between students’ engagement in STEM at secondary-school and their creativity levels (measured by DT scores). It did this by exploring both the impact of exposure to STEM (choosing STEM subjects as optional subjects/s) and the enjoyment of STEM (considering STEM subjects as a favourite subject/s). 

Guided by the extant literature, a parsimonious model was employed that sought to predict creativity by age, gender, parental education, and involvement in creative activities at school and during free time. Subsequently, the model was re-estimated with additional test variables that captured students’ exposure to STEM and their enjoyment of STEM subjects. Our key finding is that both exposure to STEM as optional subject/s and enjoyment of STEM as a favourite subject/s significantly and positively predict creativity outcomes, thus offering more support for our hypotheses. We believe that these findings contribute to the literature on the links between STEM and the development of transversal skills (e.g., Froschauer 2015; Sánchez et al. 2022; Stylianidou et al. 2018; Zhbanova 2019). Furthermore, they support the previous research on the complementarity of STEM and creativity (e.g., Root-Bernstein et al. 2008; Sánchez et al. 2022; Schoevers et al. 2020; Stylianidou et al. 2018; Zhbanova 2019). 

Further detailed regression analyses revealed that exposure to STEM as optional subjects was significantly associated with overall creativity, elaboration, originality, and novelty, but not with fluency, flexibility, or relevance. On the other hand, students who enjoyed STEM subjects—so much that they considered them their favourite subject/s—demonstrated significantly higher creativity levels for all of the six indices (fluency, flexibility, elaboration, originality, novelty, and relevance). Intrinsic motivation—a factor linked with creativity (Amabile 1996) could be at play. The finding hints at the importance of schools offering relevant and motivating STEM experiences that not only entice students to choose STEM subjects, but also result in their enjoyment of these subjects. Initiatives, such as co-curricular STEM clubs within schools, allow students the flexibility to experiment, explore, and apply STEM to projects they are personally invested in. Initiatives can promote the application of STEM skills, such as science fairs and competitions. As Peppler and Wohlwend (2018) argue, creativity itself can facilitate the engagement in these subjects as it makes subjects intrinsically more approachable and relatable. 

This latter notion of engagement is also important in the discourse relating to equity. It is noteworthy that our findings reveal that girls are considerably less likely to study STEM subjects as optional or favourite subject/s in Malta. Many countries have attempted to target this gender gap in STEM education and employment. Common strategies include informal programmes targeting underrepresented groups and industry-led campaigns in formal- and informal-education settings. Initiatives to promote equitable STEM education could look at engaging females through a holistic approach, which includes different stakeholders from the public, private, academic, and tertiary sectors.

Although the relationship between studying STEM subjects and creativity clearly emerges from this study, it is important to consider some of the limitations in the method. The main limitations relate to the identification of the key variables (STEM engagement and creativity), the sampling frame, and the cross-sectional nature of the data. 

With the data available, it was not possible to control for students’ prior STEM education, nor for any differences in STEM experiences outside school. It was also not possible to control for the quality of the STEM experience that they received—the dataset did not contain any information related to the pedagogical approach in schools, nor was it possible to estimate the models for individual schools. Such issues could be tackled in future research where data collection can permit detailed questions to be asked. On the other hand, we believe that the study offers a novel contribution to distinguishing between the choice of STEM subjects as an optional subject and the enjoyment of STEM subjects as a favourite topic. 

With regards to the measurement of creativity, the study employed data that were generated using Guilford et al.’s (1960) AUT as a creativity measure. Although they are not perfectly analogous (Runco 2010), DT tests are widely used by creativity researchers (e.g., Gu et al. 2019) as they provide estimates of creative potential, ideation, and everyday problem-solving tasks. It is well known that the test does not necessarily account for all the skills related to creativity, but that it mainly reveals DT skills. The fact that the test involves a systematic enquiry might resonate more closely with the systematic thinking approach used in scientific enquiry, thereby favouring students of scientific subjects. In subsequent studies, this could be investigated by using different tests and measures of creativity.

A different set of limitations pertain to the sample. The dataset was gathered from schools that opted into a government-funded creativity programme. This suggests that they were already inclined to include topics related to creativity within their curricula or pedagogy, and that students’ exposure to creativity may be greater than the exposure of students in other schools. With this said, there was no reason for this to bias the results in relation to the differences observed between STEM students and others. A related limitation pertains to the cross-sectional nature of the data. Future research could adopt a longitudinal approach to explore the causal effects of STEM engagement on creativity over time. This, together with a more nuanced measure of STEM engagement, a wider set of measures for creativity, and a broader range of schools would constitute an improvement to this study. 

## 6. Conclusions

This study provided evidence that the study of STEM is linked with higher levels of divergent thinking. This was determined through the OLS regression analysis using a sample of 400 students aged between 11 and 16 years. Considering that schools have generally invested in developing their STEM capacity, but are often criticised for inadequately nurturing transversal skills, these findings offer encouraging insights into 21st century education, as they suggest that, in addition to having value in their own right, STEM subjects may also contribute to the development of creativity in young people.

## Figures and Tables

**Table 1:** Descriptive statistics.

	Responses (*N*)	Minimum	Maximum	Mean	Standard Deviation
**Creativity**					
Score based on AUT (DT test), PCA	391	0	1	0.5	0.1
**STEM engagement**					
**STEM exposure** (STEM optional subjects chosen)	400	0	5	1.4	1.1
**STEM enjoyment** (STEM favourite subjects)	400	0	3	0.3	0.5
**Co-determinants of creativity**					
Extent of creative activity within school (index)	400	0	14	4.3	3.2
Extent of creative activity during free time (index)	400	0	14	5.1	3.2
Parental education (0 = no tertiary education, 1 = tertiary education)	400	0	1	0.6	0.5
Age (11–16 years old)	400	11	16	13.6	0.9
Gender (0 = male, 1 = female, 2 = prefer not to answer)	399	0	2	-	-

**Table 2:** Students’ exposure to STEM subjects.

Number of STEM Subjects Chosen as Optional/s	Frequency (*n*)	Percentage (%)
0	108	27.0
1	107	26.8
2	132	33.0
3	45	11.3
4 or 5	8	2.0

Note: *N* = 400.

**Table 3:** Students’ enjoyment of STEM subjects.

Number of STEM Subjects Named as Favourite/s	Frequency (*n*)	Percentage (%)
0	259	64.8
1	135	33.7
2 or 3	6	2.5

Note: *N* = 400.

**Table 4:** Spearman’s rank order correlation matrix.

	1	2	3	4	5	6	7
1. Age							
2. Gender	−.24						
3. Parental education	−.154 **	−.041					
4. Extent of creative activity within school	−.191 **	.019	.045				
5. Extent of creative activity during free time	−.197 **	.026	.104 *	.377 **			
6. STEM exposure (STEM optional subjects chosen)	−.205 **	-.286 **	.365 **	.075	.157 **		
7. STEM enjoyment (STEM favourite subjects)	.096	−.271 **	.134 **	-.192 **	.003	.349 **	
8. Creativity (AUT PCA)	−.069	−.185 **	.239 **	.160 **	.211 **	.264 **	.159 **

Note: *N* = 400; * *p* < .05, ** *p* < .01.

**Table 5:** OLS regression: predicting individual creativity scores (PCA).

	Base Model	H1a (STEM Optional)	H1b (STEM Favourite)
	Coeff. [SE]	Coeff. [SE]	Coeff. [SE]
Age	−0.002 [0.008]	0.001 [0.008]	−0.004 [0.008]
Gender	−0.041 ** [0.013]	−0.032 * [0.013]	−0.033 * [0.013]
Parental education	0.032 *** [0.008]	0.026 ** [0.008]	0.029 *** [0.008]
Creative activity in school	0.003 [0.002]	0.003 [0.002]	0.004 [0.002]
Creative activity in free time	0.007 ** [0.002]	0.006 ** [0.007]	0.006 ** [0.002]
STEM exposure		0.017 * [0.007]	
STEM enjoyment			0.038 * [0.015]
Constant	0.463 *** [0.113]	0.390 ** [0.116]	0.462 *** [0.112]
*R*-squared	0.120	0.135	0.135
*F*	10.486 ***	9.895 ***	9.914 ***

Note: coefficients are shown with standard errors in parenthesis. *** *p* < .001; ** *p* < .01; * *p* < .05; two-tailed. *N* = 400.

**Table 6:** OLS regression abridged results: predicting creativity by STEM engagement.

	STEM Exposure Coeff. [SE]	STEM Enjoyment Coeff. [SE]
Fluency	0.016 [0.011]	0.055 * [0.023]
Flexibility	0.018 † [0.010]	0.052 * [0.022]
Elaboration	0.022 † [0.013]	0.074 ** [0.028]
Originality	0.045 *** [0.017]	0.053 † [0.030]
Novelty	0.037 * [0.015]	0.089 ** [0.038]
Relevance	0.014 [0.015]	0.065 * [0.033]

Note: † *p* < .10; * *p* < .05; ** *p* < .01; *** *p* < .001; two-tailed; *N* = 400. Full models are available from the authors upon request.

## Data Availability

The study utilised secondary data Requests for access to the original data may be made via the corresponding author.
